# *Leptospira borgpetersenii* serovar Hardjo and *Leptospira santarosai* serogroup Pyrogenes isolated from bovine dairy herds in Puerto Rico

**DOI:** 10.3389/fvets.2022.1025282

**Published:** 2022-11-17

**Authors:** Camila Hamond, Katherine L. Dirsmith, Karen LeCount, Fred V. Soltero, Sarai Rivera-Garcia, Patrick Camp, Tammy Anderson, Jessica A. Hicks, Renee Galloway, Graham Sutherland, Ilana J. Schafer, Marga G. A. Goris, Hans van der Linden, Tod Stuber, Darrell O. Bayles, Linda K. Schlater, Jarlath E. Nally

**Affiliations:** ^1^National Veterinary Services Laboratories, Animal and Plant Health Inspection Service (APHIS), U.S. Department of Agriculture, Ames, IA, United States; ^2^National Center for Animal Health Leptospira Working Group, U. S. Department of Agriculture, Ames, IA, United States; ^3^Animal and Plant Health Inspection Service, U.S. Department of Agriculture, Veterinary Services Field Operations, San Juan, Puerto Rico; ^4^Bacterial Special Pathogens Branch, Division of High-Consequence Pathogens and Pathology, Centers for Disease Control and Prevention, Atlanta, GA, United States; ^5^Department of Medical Microbiology and Infection Prevention, World Organisation for Animal Health and National Collaborating Centre for Reference and Research on Leptospirosis, Amsterdam University Medical Center, University of Amsterdam, Amsterdam, Netherlands; ^6^Infectious Bacterial Diseases Research Unit, Agricultural Research Service, United States Department of Agriculture, Ames, IA, United States

**Keywords:** *Leptospira*, *borgpetersenii*, *santarosai*, leptospirosis, cow, dairy, Puerto Rico

## Abstract

Leptospirosis is one of the most common zoonotic diseases in the world and endemic in the Caribbean Islands. Bovine leptospirosis is an important reproductive disease. Globally, cattle are recognized as a reservoir host for *L. borgpetersenii* serovar Hardjo, which is transmitted *via* urine, semen, and uterine discharges, and can result in abortion and poor reproductive performance. The dairy industry in Puerto Rico comprises up to 25% of agriculture-related income and is historically the most financially important agricultural commodity on the island. In this study, we report the isolation of two different pathogenic *Leptospira* species, from two different serogroups, from urine samples collected from dairy cows in Puerto Rico: *L. borgpetersenii* serogroup Sejroe serovar Hardjo and *L. santarosai* serogroup Pyrogenes. Recovered isolates were classified using whole-genome sequencing, serotyping with reference antisera and monoclonal antibodies, and immunoblotting. These results demonstrate that dairy herds in Puerto Rico can be concurrently infected with more than one species and serovar of *Leptospira*, and that bacterin vaccines and serologic diagnostics should account for this when applying intervention and diagnostic strategies.

## Introduction

Leptospirosis is a worldwide zoonotic disease with an estimated 1.03 million cases and 58,900 human deaths annually (1). Leptospirosis is endemic in the Caribbean islands including Jamaica, Martinique, Haiti, Trinidad and Tobago (2, 3), and the U.S. Virgin Islands (4–6). Leptospirosis is increasingly being diagnosed after hurricane events in Central America and Puerto Rico (7, 8). In Puerto Rico, the incidence and prevalence of human leptospirosis is largely underestimated since clinical signs are associated with other febrile diseases, including dengue, malaria, and Zika (8, 9). A total of 93 leptospirosis cases in humans were reported in Puerto Rico in 2019 (10). The most frequent reactive serogroups among human patients were Icterohaemorrhagiae, Autumnalis, Mini, Ballum, Australis, Bataviae, and Canicola (8, 11). Analysis of environmental samples in Puerto Rico has confirmed the presence of multiple and diverse species of leptospires, including a recently identified new pathogenic species that represents a novel serogroup (12, 13).

Bovine leptospirosis can cause abortion, infertility, stillbirths, weak offspring, and decreased milk production. Globally, cattle are recognized as a reservoir host for *L. borgpetersenii* serovar Hardjo (14). The dairy industry in Puerto Rico comprises up to 25% of agriculture-related income and is historically the most financially important agricultural commodity on the island (15). Livestock farming and associated abattoir workers have occupational risk factors for exposure to *Leptospira* species from cattle due to shared environments and high levels of animal contact (16, 17). Multiple species, including *L. interrogans, L. kirschneri, L. borgpetersenii, L. santarosai*, and *L. noguchii*, are associated with bovine leptospirosis in South America (18–23); *L. kirschneri* and *L. santarosai* in Mexico (24) and *L. interrogans, L. kirschneri* and *L. borgpetersenii* in the U.S. (25–27).

Though the culture of pathogenic *Leptospira* from animals is inherently difficult due to their fastidious growth requirements (28), a recovered isolate is essential for accurate epidemiology and the ability to perform comprehensive genome analysis and serotyping (25, 29, 30). This in turn informs better diagnostics and bacterin vaccine strategies. Here, we describe the isolation and characterization of both *L*. *borgpetersenii* and *L*. *santarosai* from dairy cows in Puerto Rico.

## Materials and methods

### Sample collection

USDA Veterinary Services staff in Puerto Rico operated an approximately year-long tick treatment program in cattle in response to the discovery of a multi-acaricide resistant tick (*Rhipicephalus microplus*). Response activities included tick treatment of cattle at regular intervals at various dairy farms throughout Puerto Rico. During these treatment visits, opportunistic sampling for leptospirosis was performed in which free catch urine samples were collected on a selected number of animals (*N* = 49) as time and weather conditions allowed. These samples were then sent to the National Center for Animal Health (NCAH) *Leptospira* working group in Ames, Iowa, for PCR and fluorescent antibody testing (FAT) as previously described (4, 6).

Out of those animals that yielded PCR-positive urine samples, two to three animals from each farm were selected for urine sample collection to be used for culture of leptospires. In addition, a serum sample was collected for the microscopic agglutination test (MAT). The farm owners were contacted and informed of positive PCR results and were asked to separate the two to three pre-selected, PCR-positive animals from the rest of the herd to facilitate urine sample collection. On the day of sampling, the selected cows were moved into an alley or chute one by one. For each animal sampled, an assistant held the tail up and to the side. A 30 cc syringe was filled with sterile water and was used to thoroughly clean the vulva and surrounding area to remove all visible debris. Clippers were used to remove hair from the vulvar region. The vulvar region was again washed with sterile water. If any debris remained, a sterile alcohol wipe (soaked with 70% EtOH) was used to remove debris, and the wash with sterile water was repeated. The vulvar region was then sprayed with 70% EtOH. This process was often repeated multiple times to clean the area. The sample collector then stimulated the cow to urinate by rubbing the region distal to the vulva. In the other hand, which remained clean, a sterile specimen collection cup was held below the vulva and a first void urine sample was collected, which contained ~200 mL of urine. The sample collector then used a new, sterile specimen collection cup to collect a second void of urine. Then, the sample collector switched gloves and inoculated culture media with each void of urine. For this process, a sterile pipette was used to transfer 1 mL of urine from the first void sample into each of three conical tubes containing 9 mL of HAN transport media. The same process was followed for inoculation of the transport media with the second void urine sample. Any remaining urine from each void was transferred to a 50 mL conical tube, and the lid was sealed with parafilm and submitted for PCR analysis.

Samples were collected from two dairy cow farms in Puerto Rico. The first farm was in Yabucoa and although exact numbers of cattle present at the farm at any given time varied during the year-long tick treatment program, this farm comprised 629 dairy cattle at the end of the treatment program. Additionally, there were 145 beef cattle and 11 horses also housed at this dairy farm at the end of the program. The second farm was in Quebradillas and at the end of the treatment period, comprised 349 dairy cows. Animals at either farm had not been vaccinated against leptospirosis and did not show any clinical signs compatible with leptospirosis as a reproductive problem.

### Microscopic agglutination test

The microscopic agglutination test (MAT) was performed using a panel of 18 antigens representative of 15 serogroups (Supplementary Table 1). A titer was considered positive at ≥1:100 (31).

### FAT and PCR

A 45 mL aliquot of urine was centrifuged at 10,000 × *g* for 30 min at 4°C. The supernatant was removed, and the resultant pellet was resuspended in 1 mL PBS. The resuspended sample was centrifuged at 12,000 × *g* for 10 min at 4°C. The supernatant was removed until approximately 150 μL remained and this was resuspended in 1 mL PBS. The pellet was again harvested by centrifugation at 12,000 × *g* for 10 min at 4°C. The supernatant was removed until ~150 μL remained.

A 10 μL aliquot of the 150 μL that remained was placed on a glass slide within a 7 mm well, in duplicate, and FAT was performed as previously described (27). DNA was extracted from the remaining sample using the Maxwell RSC Purefood Purification Pathogen kit (Promega Corporation, Madison, Wisconsin, USA), following manufacturer's instructions, but using 1 h of incubation with 200 μL lysis buffer A and a 100 μL elution volume. PCR for *lipL32* was performed as previously described (25, 32, 33).

### Culture

A 1 mL aliquot of freshly collected urine was immediately inoculated into 9 mL of transport HAN media which was transported by overnight delivery services at ambient temperature to the National Animal Disease Center, USDA, Ames, Iowa. On arrival, a 200 μL aliquot of inoculated transport HAN media was used to inoculate 5 mL HAN semi-solid and 5 mL T80/40/LH semi-solid media. Inoculated T80/40/LH media was incubated at 29°C and inoculated HAN media was incubated at both 29°C without CO_2_ as well as 37°C in 3% CO_2_ (28, 34). Semi-solid cultures were observed using a lighted black background to examine for the appearance of a Dinger's zone (DZ), and if noted, were confirmed as positive by dark-field microscopy (DFM), at days 3 and 5, weekly for 1 month, and monthly thereafter for 6 months.

### Serological and molecular typing of *Leptospira* isolates

Cultured bovine urine isolates of *Leptospira* were serotyped by the MAT method using a panel of polyclonal rabbit reference antisera representing thirteen serogroups; Australis, Autumnalis, Ballum, Bataviae, Canicola, Grippotyphosa, Hebdomadis, Icterohaemorrhagiae, Mini, Pomona, Pyrogenes, Sejröe, and Tarassovi (National Veterinary Services Laboratories, APHIS, USDA, Ames, Iowa) (Supplementary Table 2). The isolates were further typed to the serovar level by performing MAT with panels of monoclonal antibodies (mAbs) that characteristically agglutinate serovars from the serogroup Sejroe and Pyrogenes as previously described (35).

For Illumina Sequencing, DNA was extracted from independent 5 mL cultures of each isolated strain using the Maxwell RSC Purefood Purification Pathogen kit (Promega Corporation, Madison, WI), following manufacturer's instructions. For Nanopore Sequencing, DNA was extracted from independent 5 mL culture of the same three strains using the Nanobind CBB Big DNA Kit—Beta Handbook v1.8 (07/2019) (Circulomics, Baltimore, MD). The genomic DNA concentration for all preparations was determined by Qubit (Qubit dsDNA Broad Range Assay Kit, Qubit 3.0 fluorometer, Invitrogen, Carlsbad, CA, USA) to ensure that there was a minimum of 25 ng/μL for Nanopore and 1 ng/μL for Illumina sequencing. Genomic DNA purity was assessed using the NanoPhotometer Pearl^®^ (IMPLEN).

Illumina whole-genome sequence was obtained (MiSeq Desktop Sequencer, 2x250 v2 paired-end chemistry and the Nextera XT DNA Library Preparation Kit, Ilumina, San Diego, CA, USA) per manufacturer's instructions. Prior to Nanopore sequencing, purified DNA was passed through the Circulomics Short Read Eliminator Kit XS following manufacturer's instructions. DNA was again quantified using the Qubit dsDNA Broad Range Assay Kit and 1 μg was used. The Native barcoding genomic DNA Kit was used following the manufacturer's instructions. Samples were pooled in equal amounts and loaded onto a Nanopore flowcell FLMIN106. The flowcell was run for 12 h.

Illumina sequencing reads for each isolate were mapped to the reference genome *L. borgpetersenii* serovar Hardjo and *L. santarosai* serovar Alexi using the Burrows Wheeler Aligner (BWA) and Genome Analysis Toolkit (GATK); according to GATK best practices. Illumina WGS reads were taxonomically identified using Kraken (36) and visually displayed with a Krona graph (37). Reads were assembled with SPAdes (38) and verified by comparing the expected genome size with the total assembly and verifying contigs as *Leptospira* by BLAST (39) against the nucleotide database. The Nanopore sequence was processed using Guppy to perform basecalling from the fast5 files generated by the Minion. QCAT was used to demultiplex the pooled samples by barcode. Porechop was run on the demultiplexed samples to remove the nanopore adaptors from each sample. Unicycler was then used in conservative mode to perform a hybrid genome assembly from the Illumina MiSeq and Nanopore Minion data (40). The genome annotation was completed by the NCBI Prokaryotic Genome Annotation Pipeline (41).

Using kSNP3.0 (42), a reference-free phylogenetic analysis tool, assembled genomes were compared using the output maximum likelihood tree. The sequences used were downloaded from NCBI or from the NVSL in-house sequence repository. Sequences from NCBI are indicated by the accession number, while the NVSL in-house sequences are identified only by the species, serogroup, serovar, and strain.

### Gel electrophoresis and immunoblots

Leptospires (mid-late log phase, 1–3 × 10^8^ leptospires/mL) were harvested by centrifugation (10,000 × *g*, 4°C, 30 min), washed twice with PBS, and processed for one-dimensional (1-D) SDS-PAGE on 12% acrylamide gels (BioRad) as per manufacturer's guidelines. Proteins were visualized by staining with Sypro Ruby (Invitrogen, CA, USA) and lipopolysaccharide was visualized by staining with Pro-Q Emerald 300 (Invitrogen, CA) as per manufacturer's guidelines. For immunoblotting, samples were transferred by semi-dry transfer (Amersham TE77 PWR) to Immobilon-P transfer membrane (Millipore, 220 Bedford, MA) and blocked overnight at 4°C with Starting Block (PBS) blocking buffer (Thermo Scientific, CO) (25).

Membranes were individually incubated with indicated antisera diluted in blocking buffer (anti-LipL32 at 1:4,000, or anti-Alexi, anti-Hardjo at 1:1,000) followed by incubation with horseradish-peroxidase anti-rabbit immunoglobulin G conjugate diluted 1:4,000 in blocking buffer (Sigma, MO). Bound conjugates were detected using Clarity Western ECL substrate (BioRad, CA) and images acquired using a Bio-Rad ChemiDoc MP imaging system.

## Results

### Screening for bovine urine *lipL32* PCR positive samples

In our prescreen, 7/35 (20%) bovine urine samples from farm 1, and 3/14 (21.4%) bovine urine samples from farm 2, were positive for *Leptospira* by PCR. By FAT, 2/35 (5.7%) and 1/14 (7.1%) bovine urine samples were positive on farm 1 and farm 2, respectively. All samples positive by FAT were also positive by PCR. Three PCR-positive animals on farm 1, and two PCR-positive animals on farm 2, were selected for further sampling to facilitate culture (Table 1). Of these repeat samples, three (100%; 3/3) urine samples on farm 1 (DCP-009, DCP-017 and DCP-026) were PCR positive in both voids but only one (50%; 1/2) urine sample from farm 2 (DCP-041) was positive in both voids (Table 1).

**Table 1:** Detection of *Leptospira* in urine by PCR and culture.

**Farm**	**Animal ID**	**MAT**	**PCR (prescreen)**	**PCR (Sample for culture)**		**Culture**			***Leptospira*** **isolates**		
				**Void**	**Result**	**HAN SS 29°C^a^**	**HAN SS 37°C^b^**	**T80/40/LH^c^**	**Species**	**Serogroup**	**Serovar**
Farm 1 (Yabucoa)	DCP-009	1:100 Sejroe	Positive (Ct 29.9)	1	Positive (Ct 30.9)	Negative	Negative	Negative	*L. borgpetersenii*	1:25,600 Sejroe	Hardjo
				2	Positive (Ct 30.5)	Negative	10	Negative			
	DCP-017	1:200 Australis	Positive (Ct 28.3)	1	Positive (Ct 38.7)	Negative	Negative	Negative	*L. santarosai*	1:1,600 Pyrogenes	Not defined
				2	Positive (Ct 37.4)	Negative	15	Negative			
	DCP-026	Negative	Positive (Ct 36.8)	1	Positive (Ct 36.3)	Negative	Negative	Negative			
				2	Positive (Ct 35.9)	Negative	Negative	Negative			
Farm 2 (Quebradillas)	DCP-041	1:200 Sejroe	Positive (Ct 31.4)	1	Positive (Ct 35.6)	Negative	30	30	*L. borgpetersenii*	1:12,800 Sejroe	Hardjo
				2	Positive (Ct 34.3)	25	19	25			
	DCP-046	Negative	Positive (Ct 30)	1	Negative	Negative	Negative	Negative			
				2	Negative	Negative	Negative	Negative			

^a^Number indicates days post-inoculation that evidence of a Dinger's zone was confirmed by DFM in HAN semi-solid media at 29°C.

^b^Number indicates days post-inoculation that evidence of a Dinger's zone was confirmed by DFM in HAN semi-solid media at 37°C.

^c^Number indicates days post-inoculation that evidence of a Dinger's zone was confirmed by DFM in T80/40/LH semi-solid media.

A serum sample collected at the same time as urine samples for culture showed that only 3/5 animals had a positive MAT titer: On farm 1, DCP-009 had a titer of 1:100 to serogroup Sejroe and DCP-017 had a titer of 1:200 to serogroup Australis while on farm 2, DCP-041 had a titer of 1:200 to serogroup Sejroe (Table 1).

### Culture

On farm 1, two animals were culture positive (DCP-009 & DCP-017) and one animal was culture positive on farm 2 (DCP-041). A single positive culture from the second void of DCP-009 and DCP-017 in farm 1 samples was obtained in HAN media incubated at 37°C in 3% CO_2_, but both samples were negative in HAN incubated at 29°C. In farm 2 samples, both void 1 and void 2 from DCP-041 were culture positive in HAN incubated at 37°C in 3% CO_2_, and in T80/40/LH incubated at 29°C; void 2 also was culture positive in HAN incubated at 29°C (Table 1).

### Typing of *Leptospira* isolates

**Figure 1:**
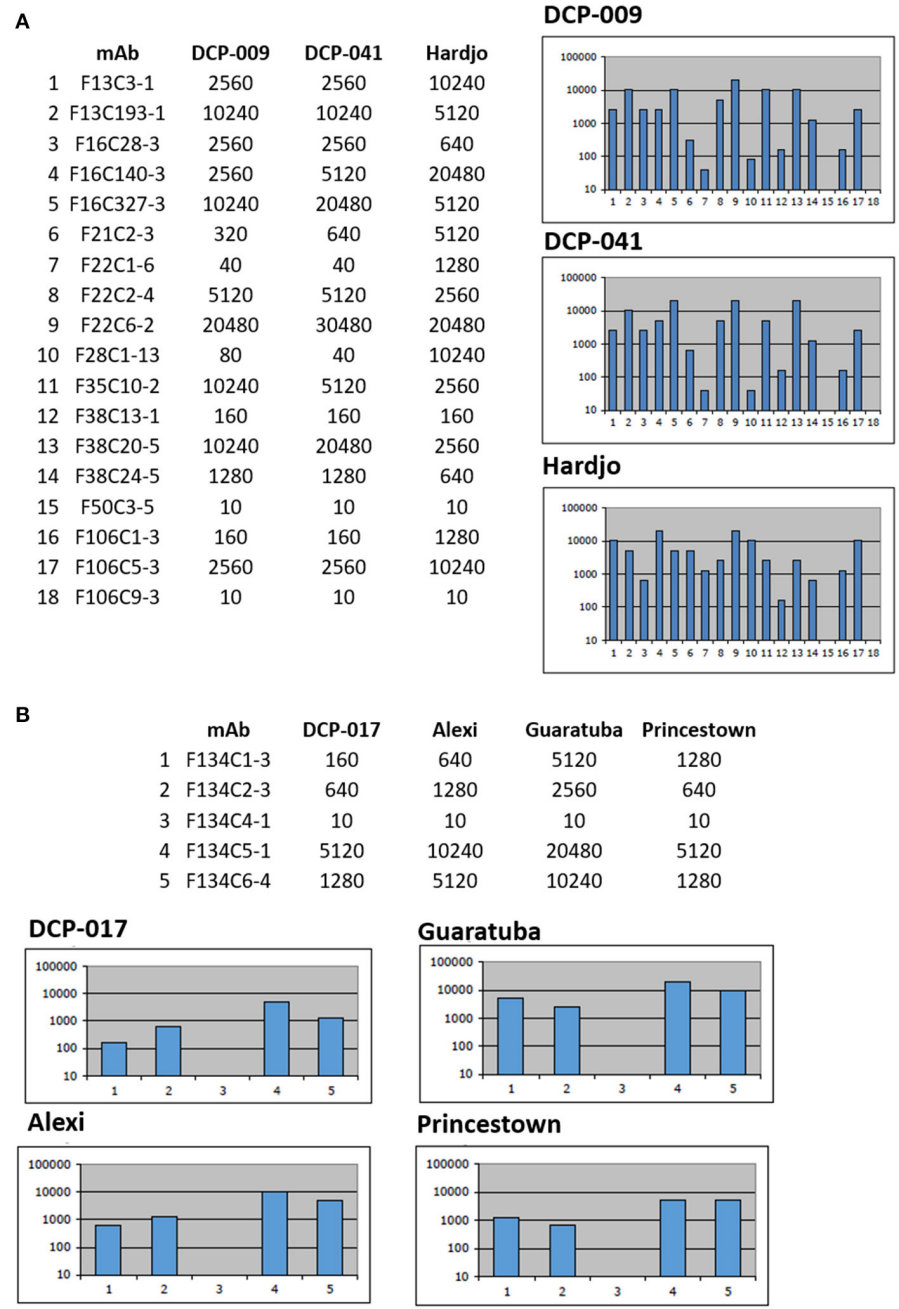
Serotyping with monoclonal antibodies (mAb) that characteristically agglutinate serovars from the serogroup Sejroe and serogroup Pyrogenes. Titers of reactivity for each mAb are provided for **(A)** *L*. *borgpetersenii* strains DCP-009, DCP-041 and reference strain *L*. *interrogans* serogroup Sejroe serovar Hardjo strain Hardjoprajitno and **(B)** *L*. *santarosai* strain DCP-017 and reference strains for serogroup Pyrogenes; *L*. *santarosai* serovar Alexi strain HS 616, *L*. *interrogans* serovar Guaratuba strain An 775 and *L*. *santarosai* serovar Princestown strain TRVL 112499. Reciprocal titers are shown on the *y*-axis; mAb number is shown on the *x*-axis.

Serotyping of strains DCP-009 and DCP-041 by MAT with reference antisera against serogroups indicated that both belong to serogroup Sejroe, but strain DCP-017 belongs to serogroup Pyrogenes, Table 1. Additional serotyping with monoclonal antibodies to identify serovar confirmed that strains DCP-009 and DCP-041 belonged to serogroup Sejroe serovar Hardjo, due to their similar reactivity patterns with the Hardjo reference strain (Figure 1A). A serovar determination for strain DCP-017 was not possible as serotyping with monoclonal antibodies was unable to discriminate whether it belonged to serovar Alexi, Guaratuba or Princestown (Figure 1B).

Molecular typing indicated that strain DCP-009 and strain DCP-017 cultured from cows on farm 1 were *L. borgpetersenii* and *L. santarosai*, respectively. Strain DCP-041 cultured from farm 2 was *L. borgpetersenii*. Accession numbers for chromosome 1 and chromosome 2 for each of the three strains, as well as genome annotation features, are provided in Table 2.

**Table 2:** Genome annotation of strains DCP-009, DCP-017 and DCP-041.

	**DCP-009**	**DCP-017**	**DCP-041**
**Species**	***L. borgpetersenii***	***L. santarosai***	***L. borgpetersenii***
Accession Number (Chromosome 1 & 2)	CP097243	CP097245	CP096186
	CP097244	CP097246	CP096185
Chromosome 1 (bp)	3,585,524	3,744,822	3,583,958
Chromosome 2 (bp)	317,338	361,033	317,340
G + C %	40.2	41.7	40.2
Genes (total)	3,387	3,742	3,399
CDSs (total)	3,343	3,698	3,355
Genes (coding)	2,944	3,601	2,949
CDSs (with protein)	2,944	3,601	2,949
Genes (RNA)	44	44	44
rRNAs (5S, 16S, 23S)	1, 2, 2	1, 2, 2	1, 2, 2
tRNAs	37	37	37
ncRNAs	2	2	2
Pseudo Genes (total)	399	97	406

**Figure 2:**
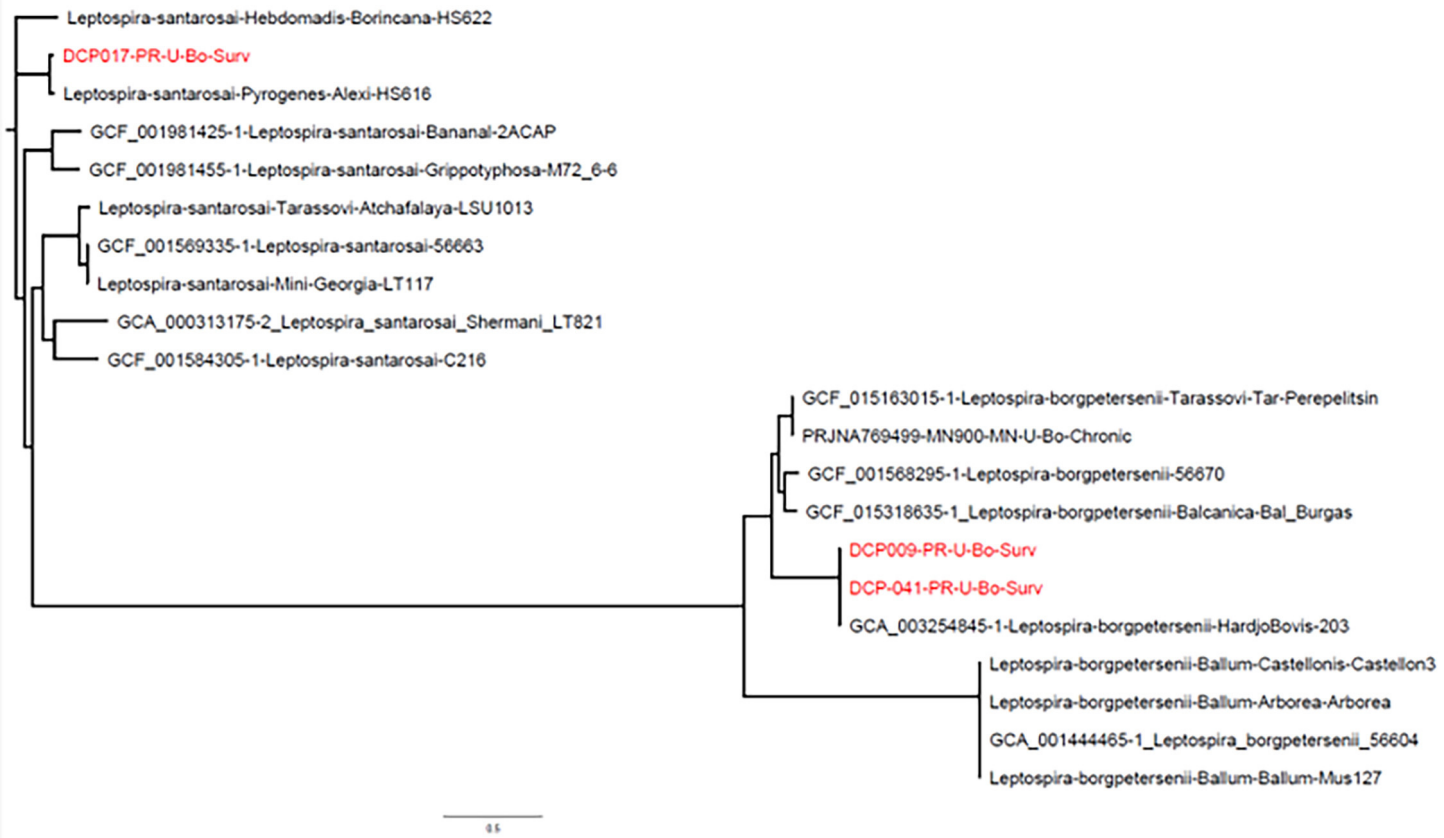
Phylogeny of *L. borgpetersenii* strain DCP-009 and strain DCP-041 and *L. santarosai* strain DCP-017 based on complete whole genome sequence analysis. Genome sequences from GenBank are preceded by an accession number, while the NVSL in-house sequences are identified only by the species, serogroup, serovar, and strain.

Phylogenetic analysis based on complete whole genome sequence demonstrates clustering of DCP-009 and DCP-041 with *L. borgpetersenii* and that strain DCP-017 clusters with *L. santarosai* (Figure 2).

### Proteins and lipopolysaccharide

**Figure 3:**
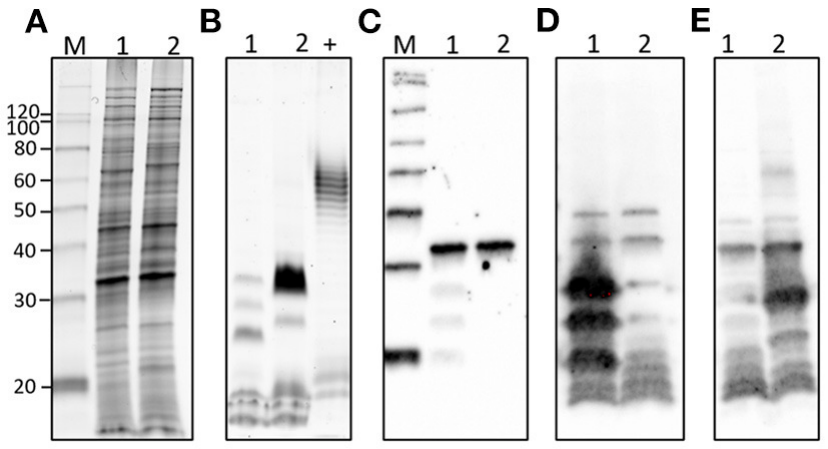
Representative images of (1) *L*. *borgeptersenii* serovar Hardjo strain DCP-009 and (2) *L*. *santarosai* serogroup Pyrogenes strain DCP-017 showing **(A)** total protein profiles, **(B)** total lipopolysaccharide profiles, **(C)** immunoblotting with anti-LipL32, **(D)** immunoblotting with anti-serovar Hardjo and **(E)** immunoblotting with anti-serovar Alexi. Five microgram of each strain was loaded per lane. +ve; postive control for LPS staining comprising 5 μg of *E*. *coli* serotype 055:B5. Molecular mass markers are indicated.

*L*. *borgpetersenii* serogroup Sejroe serovar Hardjo strain DCP-009 and *L*. *santarosai* serogroup Pyrogenes strain DCP-017 have a similar protein profile (Figure 3A) and both express the pathogen-associated outer membrane protein LipL32 (Figure 3C). In contrast, and as expected with strains of pathogenic leptospires belonging to different serogroups and serovars, each strain presented with a unique lipopolysaccharide profile (Figure 3B) as confirmed by immunoblotting with antisera specific for serovar Hardjo and serovar Alexi (Figures 3D,E, respectively).

## Discussion

There is limited understanding of bovine leptospirosis in Puerto Rico. This study cultured and characterized isolates of *Leptospira* from dairy cows in Puerto Rico to determine what species and serovars are circulating in local dairy herds to aid in developing vaccines and diagnostics, and to understand the zoonotic risks of disease transmission.

*L. borgpetersenii* serovar Hardjo was isolated from two different dairy herds in Puerto Rico: though these represent the first serovar Hardjo isolates from dairy cows in Puerto Rico to our knowledge, it is consistent with the global distribution of leptospirosis in bovines which act as reservoir hosts for *L. borgpetersenii* serovar Hardjo, often with no apparent clinical signs of infection (14). In addition, *L. santarosai* serogroup Pyrogenes was also isolated in Puerto Rico, and from the same dairy herd shedding *L. borgpetersenii* serovar Hardjo. We were unable to serotype *L*. *santarosai* serogroup Pyogenes strain DCP-017 to the serovar level since monoclonal antibodies could not distinguish whether it belonged to serovar Alexi, Princestown, or Guaratuba. However, the reference strains for serovar Alexi and Princestown are also *L*. *santarosai* compared to serovar Guaratuba which belongs to the species *L*. *interrogans*. Serovar Alexi was originally isolated from a human patient in Puerto Rico in 1951 and, after comparison with serovars Pyrogenes and Zanoni (both of the serogroup Pyrogenes), the reference strain HS 616 was recognized as a new serovar (43). This new serovar first appears in the WHO list of 1965 and the strain was submitted to factor analysis by Kmety (44), who confirmed its status and thus inclusion in the subgroup Pyrogenes (45). Serovar Princestown was originally isolated from the blood of a 15-year-old boy from Princestown, Trinidad, West Indies, and described as a new serovar named Princestown, reference strain TRVL 112499, in the Pyrogenes group (46). In contrast, serovar Guaratuba reference strain An 7705 was originally isolated from an opossum in Brazil (47). Collectively, this would suggest the isolate from DCP-017 is more likely closer to serovar Alexi. Two strains are said to belong to different serovars if, after cross absorption with adequate amounts of heterologous antigen, more than 10% of the homologous titer regularly remains in at least one of the two antisera in repeated tests (48); the cross agglutination absorption test (CAAT) is required to determine if strain DCP-017 represents a new serovar (45).

*L. santarosai* is commonly associated with infection of humans, and domestic and wildlife animals in Latin America. This species has been isolated from cattle in Brazil (18, 20–22), Mexico (24), and Peru (23). Recently, *L. santarosai* was isolated from the uterus of a sub-fertile cow, highlighting a potential role in bovine genital leptospirosis and poor reproductive performance (21, 49). Clinical symptoms of human patients infected with *L. santarosai* range from mild to severe, including Weil's syndrome and liver failure (50). This coupled with the isolation of *L. santarosai* serovar Alexi strain HS 616 (serogroup Pyrogenes) and *L. santarosai* serovar Borincana strain HS622 (serogroup Hebdomadis) from human patients in Puerto Rico (43) highlights the zoonotic risk of infection.

Culture of leptospires provides a definitive diagnosis as well as an isolate that can be completely characterized at the genotypic and phenotypic level, included in an MAT diagnostic antigen rack, and potentially used for bacterin-based vaccination (51). Culture of leptospires is a technically difficult and laborious task but the detection of PCR-positive dairy cows prior to culture provides a screening tool to prioritize efforts and optimize successful outcomes. The recent development of new media formulations to support the growth of fastidious leptospires has enabled collection of samples which can now be transported long distances prior to processing (5, 6, 28). Both *L*. *borgpetersenii* and *L*. *santarosai* have similar protein profiles and express the pathogen-associated outer membrane protein LipL32. The characterization of these strains to serovar level highlights the significantly different expression of lipopolysaccharide (LPS). The O-antigen of LPS is a protective antigen and thus our results provide insight into which serovars should be considered for more efficacious bovine vaccine strategies, to limit animal disease as well as zoonotic transmission.

In this study, both cows (DCP-009 and DCP-041) shedding serovar Hardjo were seropositive by MAT, but the cow (DCP-017) shedding serogroup Pyrogenes was seronegative for serogroup Pyrogenes. Though seronegative animals can shed leptospires (27, 52), the efficacy of the MAT is based in part on inclusion of appropriate serovars representative of each serogroup within a geographical locale. In addition, as recommended by the WOAH-Manual, the sensitivity of the MAT can be improved by using local isolates. The use of local isolates from Puerto Rico can be used on larger seroprevalence studies to determine levels of exposure by dairy cows to pathogenic leptospires.

The dairy industry is the most financially important agricultural commodity in Puerto Rico (15). Since bovine leptospirosis can result in significant economic costs (53), it is important to accurately define the epidemiological aspects of this disease to ensure efficacious intervention strategies, and to determine whether to target transmission of disease within the herd, from other domestic animals (54), or invasive small mammals (16). Our results demonstrate that both *L*. *borgpetersenii* serovar Hardjo and *L*. *santarosai* serogroup Pyrogenes infect dairy cows in Puerto Rico and highlight the need to consider multiple species and serovars to mitigate domestic animal infection and limit zoonotic transmission of leptospirosis.

## Data availability statement

The datasets presented in this study can be found in online repositories. The names of the repository/repositories and accession number(s) can be found below: https://www.ncbi.nlm.nih.gov/genbank/, CP097243, CP097244, CP097245, CP097246, CP096186, CP096185.

## Ethics statement

All sample collection was conducted in accordance with protocols as reviewed and approved by the Animal Care & Use Committee at the CDC, protocol 2879SALMULX-A3. Informed consent was obtained from the owners for the participation of their animals in this study.

## Author contributions

CH, IS, and JN: conceptualization. CH, KD, KL, FS, SR-G, PC, TA, JH, RG, GS, IS, MG, HL, TS, DB, and JN: methodology. CH, KD, KL, TA, JH, MG, HL, TS, DB, and JN: formal analysis and writing—review and editing. FS, IS, MG, and LS: resources. CH, KL, TS, MG, HL, and JN: figures. CH and JN: writing—original draft preparation. All authors have read and agreed to the published version of the manuscript.

## Funding

This research was supported by USDA and in part by an appointment to the Animal and Plant Health Inspection Service (APHIS) Research Participation Program administered by the Oak Ridge Institute for Science and Education (ORISE) through an interagency agreement between the U.S. Department of Energy (DOE) and the U.S. Department of Agriculture (USDA). ORISE is managed by ORAU under DOE contract number DE-SC0014664.

## Conflict of interest

The authors declare that the research was conducted in the absence of any commercial or financial relationships that could be construed as a potential conflict of interest.

## Publisher's note

All claims expressed in this article are solely those of the authors and do not necessarily represent those of their affiliated organizations, or those of the publisher, the editors and the reviewers. Any product that may be evaluated in this article, or claim that may be made by its manufacturer, is not guaranteed or endorsed by the publisher.

## Author disclaimer

All opinions expressed in this paper are the author's and do not necessarily reflect the policies and views of USDA, DOE, ORAU/ORISE or CDC. USDA is an equal opportunity provider and employer. Mention of trade names or commercial products in this publication is solely for the purpose of providing specific information and does not imply recommendation or endorsement by the U.S. Department of Agriculture.
